# A case of Brugada Syndrome unmasked by a postoperative febrile state

**Published:** 2015

**Authors:** Jharendra Rijal, Smith Giri, Sumesh Khanal, Khagendra Dahal

**Affiliations:** 1 Department of Internal Medicine, Staten Island University Hospital, Staten Island, 475 Seaview Avenue, Staten Island, NY 10305, USA.; 2 Department of Medicine, University of Tennesse Health Science Center, 956 Court Avenue, Memphis, TN 38163, USA.; 3 Department of Medicine, Institute of Medicine, Tribhuvan University Teaching Hospital, Maharajgunj, PO Box 1524, Kathmandu, Nepal.; 4 Department of Medicine, Lakes Region General Hospital 80 Highland Street, Laconia, NH 3246, USA.

**Keywords:** *Arrythmias*, *Brugada Syndrome*, *fever*, *Electrocardiogram*, *appendectomy*

## Abstract

**Background:**

Brugada Syndrome (BS) is an inherited ion channelopathy characterized by an electrocardiographic (ECG) pattern of a coved type ST segment elevation in right precordial leads with or without right bundle branch block.

**Case Presentation:**

A 23-year old male presented with right lower quadrant abdominal pain. Further evaluation revealed a diagnosis of acute appendicitis. The patient developed a febrile episode on second post-operative day of laparoscopic appendectomy. ECG revealed features consistent with BS. Prompt control of temperature in the patient resolved the ST-segment elevation and prevented potentially life-threatening arrhythmias.

**Conclusion:**

Febrile episodes in susceptible patients may unmask a concealed BS. Prompt control of temperature is advocated to reduce the risk of life-threatening arrythmias.

Brugada syndrome (BS) is an arrhythmogenic ion channelopathy, caused by a mutation in the cardiac sodium channels and inherited in an autosomal-dominant manner (1). Although its exact prevalence is unclear, BS is responsible for about 4% of all sudden deaths and up to 20% of sudden deaths among patients with structurally normal hearts (2). Brugada Syndrome is manifested by characteristic EKG pattern of coved ST segment elevation (≥2 mm) least two precordial leads (lead V1-V3), as well as right bundle branch block (3). A variety of triggers can unmask a concealed Brugada syndrome type EKG such as fever, electrolyte abnormalities and drugs (2, 4). This may put the patient at risk for ventricular tachyarrythmias and sudden cardiac death (2). Hence, early identification and removal of such triggers is crucial in preventing morbidity and mortality in affected patients. We present a case of 23-year-old young man with Brugada type ECG that appeared with a febrile episode on the second post-operative day of an uneventful laparoscopic appendectomy under standard anaesthesia. Prompt control of temperature in the patient resolved the ST-segment elevation and prevented potentially life-threatening arrhythmias. 

## Case Presentation

A 23-year-old male developed fever and complained of palpitations on the second post-operative day of an uneventful laparoscopic appendectomy for acute appendicitis. The patient denied any chest pain, shortness of breath, presyncopal symptoms or urinary complaints. On examination, his temperature was 102.3 F, pulse rate 120/min, respiratory rate 19/min and a blood pressure of 104.70 mmHg.

The operative site was clean. Chest examination revealed minimal crackles over the left lower base. Rest of the physical examination was normal. A bed side 12-lead ECG revealed sinus tachycardia with ST segment elevation in lead V1 to V3 with incomplete right bundle branch block (RBBB). The differential diagnosis at this time was acute myocardial infarction, acute pericarditis and BS. Cardiac markers were negative (Troponin level I <0.06 ng/ml and CK-MB 0.7 ng/ml). Complete blood count, electrolytes, blood sugar, blood urea nitrogen and D-dimer were within normal limits. 

Transthoracic echocardiogram revealed structurally and functionally normal heart. A chest x-ray was essentially normal. A computerized tomography (CT) of the chest showed minimal atelectasis of the left lower lobe. CT of the abdomen and pelvis was unremarkable. Repeat cardiac enzymes obtained 6 hours later were normal.

The fever subsided the following day and repeat ECG showed normal sinus rhythm with complete resolution of ST-segment elevation and partial RBBB. Considering the possibility of acute coronary syndrome, the patient was given 325 mg of aspirin and transferred to Coronary Care Unit (CCU). After cardiac enzymes came out to be normal, aspirin was discontinued. 

As the Brugada pattern ECG was unmasked only during febrile episode, and there were no high-risk factors, the patient was classified as having low- to intermediate- risk for arrhythmia. Therefore, he was discharged on the fifth post-op day with the knowledge about the condition and prompt management of fever. At four-weeks and three months of follow-up, the clinical condition and the ECG of the patient was unremarkable.

## Discussion

Brugada syndrome (BS) was first described as a triad of right bundle branch block pattern, ST-segment elevation and sudden cardiac death (3). With an estimated prevalence of 5 per 10,000 individuals, BS is known to cause sudden cardiac death due to its propensity to cause ventricular arrythmias (5). BS is more common among men, with a mean age at diagnosis of 40-45 years (5). Affected individuals often have a history of sudden cardiac death in the family as the inheritance is autosomal dominant (4). A variety of mutations have been linked with the syndrome; the most common being the SCN5A mutation which encodes the alpha subunit of the human cardiac voltage dependent Na channel found in as many as about 18-30% of affected patients (6). Three distinct types of ECG patterns in the right precordial leads (V_1-3_) has been described in Brugada syndrome. In the most common type 1, the coved type of ST segment elevation of ≥ 2mV gradually descends to a negative T wave, as seen in our case (7). Any condition or medication that alters the sodium currents in the heart may unmask the EKG changes in affected individuals (8). These triggers include fever, electrolyte changes, and a variety of drugs (4, 9). 

Fever has been reported to be an important trigger for unmasking of Brugada syndrome (2, 4, 6, 8, 10, 11). A rise in temperature causes inactivation of the sodium channel in some of these patients, and causes umasking of concealed Brugada syndrome, like in our patient (10). Failure of timely control of temperature can lead to life-threatening arrhythmia and cardiac arrest (11). 

Anesthetic agents can also unmask BS pattern of ECG primarily due to an effect on autonomic nervous system (12). The exposure to standard anesthetic agents in our patient did not lead to the unmasking of BS pattern of ECG or arrhythmia in our patient. This finding is an addition to a number of case reports of uneventful general anesthesia in patients with BS (-).

Definitive diagnosis of BS requires the presence of characteristic type 1 EKG changes with at least one of the following: documented ventricular arrhythmia, family history of SCD <45 yr old, coved type EKGs in family members, inducibility of ventricular arrhythmias with programmed electrical stimulation, syncope or nocturnal agonal respiration. Our patient did not have a family history of sudden cardiac death, nor was the family immediately available for ECG analysis (9). We did not perform an electrophysiological study in our patient to document the inducibility of arrythmias. 

While, ICD implantation is recommended for BS patients with high-risk for the development of ventricular arrhythmia to avert SCD; the management of low risk patients is less clear (2). A previous study suggested that there is a risk of SCDs even in low risk patients with ECG changes alone, as in our case (16). Given the inducibility of EKG changes due to fever in our patient, we advised for prompt therapy of any febrile condition to avert any possible life-threatening arrythmias. Screening of the family members was also recommended.

## References

[1] Wilde AA, Antzelevitch C, Borggrefe M (2002). Proposed diagnostic criteria for the Brugada syndrome: consensus report. Circulation.

[2] Kalra S, Iskandar SB, Duggal S, Smalligan RD (2008). Fever-induced ST-segment elevation with a Brugada syndrome type electrocardiogram. Ann Int Med.

[3] Brugada P, Brugada J (1992). Right bundle branch block, persistent ST segment elevation and sudden cardiac death: a distinct clinical and electrocardiographic syndrome. A multicenter report.. J Am Coll Cardiol.

[4] De Marco S, Giannini C, Chiavaroli V (2012). Brugada syndrome unmasked by febrile illness in an asymptomatic child. J Pediatr.

[5] Antzelevitch C, Brugada P, Brugada J (2002). Brugada syndrome: a decade of progress. Circ Res.

[6] Traub D, Fonseka N, Priori S (2011). ST-segment elevation in the setting of a febrile illness. Ann Noninvasive Electrocardiol.

[7] Berne P, Brugada J (2012). Brugada syndrome 2012. Circ J.

[8] Shalev A, Zeller L, Galante O, Shimony A, Gilutz H, Illia R (2008). Symptomatic Brugada unmasked by fever. Israel Med Assoc J.

[9] Antzelevitch C, Brugada P, Borggrefe M (2005). Brugada syndrome: report of the second consensus conference: endorsed by the Heart Rhythm Society and the European Heart Rhythm Association. Circulation.

[10] Antzelevitch C, Brugada R (2002). Fever and Brugada syndrome. Pacing Clin Electrophysiol.

[11] Gonzalez Rebollo JM, Hernandez Madrid A (2000). Recurrent ventricular fibrillation during a febrile illness in a patient with the Brugada syndrome. Rev Esp Cardiol.

[12] Hayashida H, Miyauchi Y (2006). Anaesthetic management in patients with high-risk Brugada syndrome. Br J Anaesth.

[13] Edge CJ, Blackman DJ, Gupta K, Sainsbury M (2002). General anaesthesia in a patient with Brugada syndrome. Br J Anaesth.

[14] Inamura M, Okamoto H, Kuroiwa M, Hoka S (2005). General anesthesia for patients with Brugada syndrome. A report of six cases. Can J Anaesth.

[15] Santambrogio LG, Mencherini S, Fuardo M, Caramella F, Braschi A (2005). The surgical patient with Brugada syndrome: a four-case clinical experience. Anesth Analg.

[16] Brugada P, Brugada R, Brugada J (2005). Should patients with an asymptomatic Brugada electrocardiogram undergo pharmacological and electrophysiological testing?. Circulation.

